# Dietary micronutrients intake and its effect on haemoglobin levels of pregnant women for clinic visit in the Mount Cameroon health area: a cross-sectional study

**DOI:** 10.3389/fnut.2024.1341625

**Published:** 2024-05-07

**Authors:** Vanessa Tita Jugha, Juliana Adjem Anchang, David Denis Sofeu-Feugaing, Germain Sotoing Taiwe, Helen Kuokuo Kimbi, Judith Kuoh Anchang-Kimbi

**Affiliations:** ^1^Department of Animal Biology and Conservation, University of Buea, Buea, Cameroon; ^2^International Centre for Agricultural Research in the Dry Areas, ICARDA, Cairo, Egypt; ^3^Department of Biochemistry and Molecular Biology, University of Buea, Buea, Cameroon; ^4^Department of Biomedical Sciences, University of Bamenda, Bamenda, Cameroon; ^5^Department of Microbiology and Immunology, College of Medicine, Drexel University, Philadelphia, PA, United States

**Keywords:** dietary diversity, micronutrients, haem iron, vitamin A, haemoglobin levels, pregnant women, Mt. Cameroon area, cross-sectional study

## Abstract

**Background:**

Nutritional deficiencies and its consequences such as anaemia are frequent among pregnant women residing in under resource settings. Hence, this study sought to investigate specific dietary micronutrient inadequacy and its effect on maternal haemoglobin levels.

**Methods:**

This institution based cross-sectional survey enrolled 1,014 consenting pregnant women consecutively. Data on socio-demographic, economic and antenatal characteristics were recorded using a structured questionnaire. Minimum dietary diversity for women (MDD-W) was assessed using the 24-h recall method and haemoglobin (Hb) concentration (g/dL) determined using a portable Hb metre. Significant levels between associations was set at *p* < 0.05.

**Results:**

Among those enrolled, 40.9% were anaemic while 89.6% had inadequate dietary nutrient intake. In addition, uptake of blood supplements, haem iron, plant and animal-based foods rich in vitamin A were 71.5, 86.2, 35.5 and 12.6%, respectively. Moreover, anaemia prevalence was significantly (*p* < 0.05) lower in women who took iron-folic acid along with food groups rich in haem iron (38.5%) or both plant and animal vitamin A (29.0%). Besides, mean maternal Hb levels was significantly (*p* < 0.001) higher in women who consumed haem iron (11.08 ± 1.35) and vitamin A food groups (11.34 ± 1.30) when compared with their counterparts who did not consume haem iron (10.54 ± 1.19) and vitamin A food groups (10.74 ± 1.31).

**Conclusion:**

Dietary uptake of foods rich in haem-iron and vitamin A significantly improves Hb levels in Cameroonian pregnant women. Our findings underscore the importance of improving maternal nutritional awareness and counselling during antenatal period to reduce the anaemia burden.

## Introduction

Micronutrients are vital to health as they ensure normal growth, metabolism and physical wellbeing (1, 2). Although required in small amounts, the impact of their deficiency is severe (3). Globally, more than 2 billion people suffer from micronutrient deficiencies, with the main being iron, zinc, iodine, vitamins A and B (4, 5). During pregnancy, these deficiencies which results from; lack of consumption of nutrient-dense food groups, poor understanding of the importance of a diverse diet and inefficient utilisation of available micronutrients (6, 7) can lead to a myriad of adverse maternal and perinatal outcomes including; anaemia, increased susceptibility to infectious diseases, low birth weight, preterm birth, increased risk of maternal and neonatal mortality as well as cognitive deficit in the baby later in life (2, 8).

Anaemia is a widespread public health problem that has significant consequence for human health, social development, and economic growth (9–11). According to the World Health Organization (WHO), anaemia is a condition in which the haemoglobin concentration within the red blood cells are lower than normal and consequently their oxygen carrying capacity is insufficient to meet the physiological demands of the body (12, 13). This results in symptoms such as; body weakness, fatigue, dizziness, palpitations and shortness of breath (13, 14). In 2019, the prevalence rates of anaemia was estimated at 29.9% among women of reproductive ages (WRA) and 36.5% in pregnant women (15). Though preventable, in pregnancy it is still one of the leading causes of maternal and neonatal morbidity and mortality (16, 17). Apart from nutritional deficiencies of which iron deficiency is the most prevalent cause of anaemia, other conditions such as folate, zinc, vitamin A and B deficiencies, chronic inflammation, infectious diseases and inherited haemoglobin disorders can as well lead to anaemia (12, 18, 19).

Over the past decade, awareness for anaemia and its consequences for maternal and infant health has increased. For instance, in 2012, the 65th World Health Assembly (WHA) approved global targets for maternal, infant and young child nutrition with a commitment to reduce to half the prevalence of anaemia among WRA (15–49 years) by 2025 (20, 21). Ensuing this, the WHO and United Nations Children’s Fund (UNICEF) proposed extending this target to 2030 to align with the United Nations (UN) Sustainable Development Goals (21, 22). With this in mind, Cameroon has been committed to curb the burden of maternal anaemia through malaria prophylaxis and haematinic supplementation (16). Despite efforts, anaemia prevalence rates have not changed over the years as it is still a severe (≥ 40%) health problem in WRA (23, 24). An explanation to this high prevalence rates could be an underestimation of the role of dietary micronutrient inadequacy on anaemia. Besides, data on micronutrients are limited in the study area and are thus needed, to design and implement public health programmes targeted at reducing anaemia. Hence, this study aimed to investigate intake of dietary nutrients and its effect on maternal haemoglobin levels in the Mount Cameroon health area.

## Materials and methods

### Study site

This study was conducted at the antenatal care units of various health facilities located in the Buea and Tiko Health Districts of the Mount Cameroon area. The characteristic of the study settings has been described in detail by Jugha et al. (25). More so, the different health facilities in these health districts were chosen based on their accessibility as well as the localities they serve (25, 26).

The tropical equatorial climate of the Mount Cameroon region is made up of a long rainy season accompanied by high rainfall (2,000–10,000 mm) and average temperatures conducive for agriculture, the principal economic activity in the region (27, 28). Irrespective of the agricultural biodiversity, starchy staple is the most commonly consumed food group (25). In addition, malaria is endemic in the area and transmission is perennial (29) with *Plasmodium falciparum* accounting for over 90% of malaria parasite infection (30). Also, anaemia prevalence among pregnant women (≥ 40%) over the years in the area has not changed (16, 25, 31).

### Study design, and population

This cross-sectional survey enrolled consenting pregnant women in any trimester of gestation consecutively. Study sample size was estimated using the Cochrane formulae for cross-sectional studies based on the prevalence of anaemia (40%) in the study area (25, 32). After adding for a 10% non-response rate (NRR) the overall number of women to be enrolled from both health district was 1,014.

A structured questionnaire (pre-tested) through a face-to-face interview was used to obtain maternal socio-demographic data (setting, age, marital status), educational level, household number, and antenatal clinic data (number of antenatal care visits, gestational age, parity, IPTp-SP and iron-folic acid uptake). Information relating to household wealth that is; housing type, house ownership, toilet type, possession of basic amenities (radio, car, bicycle, television, motorcycle and mobile phone) and source of drinking water were also documented. These indicators of household wealth were subjected to principal component analysis (PCA) in order to determine maternal wealth status (33).

### Dietary micronutrients assessment

The minimum dietary diversity for women (MDD-W) questionnaire, a proxy indicator of micronutrient adequacy was used to determine maternal dietary nutrient intake (34, 35). During questionnaire survey, each study respondent was requested to describe all food groups and drinks consumed day and/or night 24**-**h before the survey. These food groups (FGs) included: starchy staples; pulses; nuts and seeds; dairy; meat, poultry and fish; eggs; dark green leafy vegetables; vitamin A-rich fruits and vegetables; other vegetables and other fruits (25, 34). A score of 1 was attributed to the consumption of any food item within any food group as per the FAO guidelines (34). Dietary diversity score was obtained by summing up the FGs consumed among the 10 required FGs (34). Participants were then categorised as having adequate dietary nutrient intake if they consumed at least 5 of more food groups a day prior to the study (25, 34).

Moreover, the FGs; dark green leafy vegetables, vitamin A-rich fruits and vegetables, Meat (including organ meat), poultry, fish, eggs and milk products were further reclassified as vitamin A-rich plant foods (dark green leafy vegetables, vitamin A-rich fruits and vegetables), vitamin A-rich animal foods (organ meat, eggs and milk products) and foods rich in haem iron (meat, poultry and fish) as per the FAO guidelines (36).

### Sample collection and laboratory analysis

Venous blood (2 mL) was collected from each pregnant woman using sterile techniques. Maternal Hb concentration (g/dL) was determined in the field using a portable URIT**-**12 Hb metre (URIT Medical Electronics Co., Ltd. Guangxi, China). In this study, anaemia status was defined as Hb < 11 g/dL for gravid women in the first and third trimester and Hb < 10.5 g/dL for those in the second trimester of gestation (25, 37).

### Ethical considerations

Ethical clearance (Ref No: 2019/967-05/UB/SG/IRB/FHS) was obtained from the Faculty of Health Science Institutional Review Board (IRB), University of Buea whereas administrative authorization was gotten from the South West Regional Delegation of Public Health, District Medical and Chief Medical Officers in charge of the health districts and medical facilities, respectively. After sensitising the women on the study objectives, potential risks and benefits, those who gave their consent signed a written informed consent form and were thus included into the study whereas, those presenting with complicated pregnancy or a history of diabetes, hypertensive disorders or pre-eclampsia were not eligible to partake in the study and were therefore, excluded. In addition, participation in the study was voluntary.

### Data analysis

Data was analysed using the IBM-Statistical Package for Social Sciences (SPSS) version 23. Continuous data were checked for normality and expressed as means and standard deviation (SD). Descriptive statistics such as mean, SD, frequency and percentages were used to describe data. Furthermore, the Pearson Chi**-**square test (χ^2^) was used to evaluate the differences in proportions between uptake of iron**-**folic acid (IFA), haem iron, vitamin**-**A food groups and maternal anaemia status. In addition, comparison between the continuous variable (Hb levels) and group parameters (intake of haem iron and vitamin A food groups) was done using the student’s paired t**-**test. Statistical test was two**-**tailed and the level of significance set at *p* < 0.05.

## Results

### Characteristics of the study participants

As shown in Table 1, mean maternal age (± SD) and household size (± SD) of those enrolled was 26.72 (± 5.48) years and 4.44 (± 2.20) persons. Besides, over 50% of the women were married and had a household size of at least four and more members. Furthermore, most (33.9%) of the study participants were within the age group 25–29 years followed by those aged 19–24 years (30.4%; Table 1).

**Table 1:** Sociodemographic and economic characteristics of the women.

Variable	Total % (N)
Study site	
Tiko Health District	50.2 (509)
Buea Health District	49.8 (505)
Age (±SD) years	26.72 ± 5.48 (15–46)
15–18	5.5 (56)
19–24	30.4 (308)
25–29	33.9 (344)
30–34	20.9 (212)
≥ 35	9.3 (94)
Marital status	
Unmarried	37.8 (383)
Married	62.2 (631)
Educational level	
Below secondary	20.1 (204)
Secondary	53.1 (538)
Above secondary	26.8 (272)
Household number (± SD)	4.44 ± 2.20 (1–12)
1–3 persons	38.2 (387)
≥ 4 persons	61.8 (627)
Wealth status	
Low	56.8 (576)
High	43.2 (438)

SD, standard deviation.

### Antenatal care characteristics of the study participants

Of those enrolled, mean gestational age (± SD) was 27.60 (± 7.61) weeks. In addition, gravid women with parity 1–2 constituted 43.3% of the study population. Besides, over 70% of the women had received blood supplements in the form of iron**-**folic acid. Moreover, 35.5, 12.6 and 86.2% of the women had consumed plant foods rich in vitamin A, animal foods rich in vitamin A and haem iron, respectively (Table 2).

**Table 2:** Maternal obstetric characteristics and frequency of dietary micronutrient intake.

Variable	Total % (N)
Antenatal care visits (± SD)	2.54 ± 1.58 (1–12)
≤ 3	77.3 (784)
>3	22.7 (230)
Gestational age (± SD) weeks	27.60 ± 7.61 (6–43)
< 27	44.7 (453)
≥ 27	55.3 (561)
Parity (± SD)	1.25 ± 1.34 (0–8)
0	38.5 (390)
1–2	43.3 (439)
3–4	16.5 (167)
≥ 5	1.8 (8)
IPTp-SP uptake	
≤ 1 dose	67.7 (686)
2 doses	20.1 (204)
≥ 3 doses	12.2 (124)
Blood supplements uptake	
Yes	71.5 (725)
No	28.5 (289)
Anaemia status	
Anaemic	40.9 (415)
Non-anaemic	59.1 (599)
MDD-W (± SD)	3.57 ± 0.82 (1–7)
Adequate dietary nutrient intake	10.4 (105)
Inadequate dietary nutrient intake	89.6 (909)
Consumed plant rich vitamin A FGs	
Yes	35.5 (360)
No	64.5 (654)
Consumed animal rich vitamin A FGs	
Yes	12.6 (128)
No	87.4 (886)
Consumed haem iron	
Yes	86.2 (874)
No	13.8 (140)
Consumed plant and animal-based vitamin A FGs	
Plant based foods only	31.6 (320)
Animal based foods only	8.7 (88)
Both	3.9 (40)
None	55.8 (566)
Consumed both plant and animal vitamin A FGs	
Yes	44.2 (448)
No	55.8 (566)

MDD-W, minimum dietary diversity for women; FGs, food groups.

### Association between uptake of iron-folic acid, haem iron, vitamin A foods and maternal anaemia

As shown in Table 3, anaemia prevalence rates were lowest in women who took blood supplements (iron**-**folic acid) alongside food groups rich in haem iron (38.5%, *p* = 0.031) as well as both plant and animal vitamin A (29.0%, *p* < 0.001) when compared with their respective contemporaries who relied on IFA only (Table 3).

**Table 3:** Association between uptake of iron-folic acid, haem iron, vitamin A foods and maternal anaemia.

Factors	Categories	Total N	Anaemic % (n)	*p* value
Iron-folic acid uptake	Yes	725	40.8 (296)	0.919
	No	289	41.2 (119)	
Uptake of IFA and haem iron FGs	IFA only	108	53.7 (58)	0.031
	Haem iron FGs only	251	41.0 (103)	
	Both	623	38.5 (240)	
	None	32	43.8 (14)	
Uptake of IFA and plant Vit. A FGs	IFA only	473	46.3 (219)	0.001
	Plant vit. A FGs only	102	38.2 (39)	
	Both	258	30.6 (79)	
	None	181	43.1 (78)	
Uptake of IFA and animal Vit. A FGs	IFA only	646	43.7 (282)	< 0.001
	Animal vit. A FGs only	43	16.3 (7)	
	Both	85	18.8 (16)	
	None	240	45.8 (110)	
Uptake of IFA and combined Vit. A FGs	IFA only	417	49.6 (207)	< 0.001
	Plant and animal vit. A FGs only	134	32.1 (43)	
	Both	314	29.0 (91)	
	None	149	49.7 (74)	

IFA, iron-folic acid; FGs, food groups; Vit. A, vitamin A.

### Intake of haem iron and vitamin A food groups on haemoglobin levels

**Figure 1:**
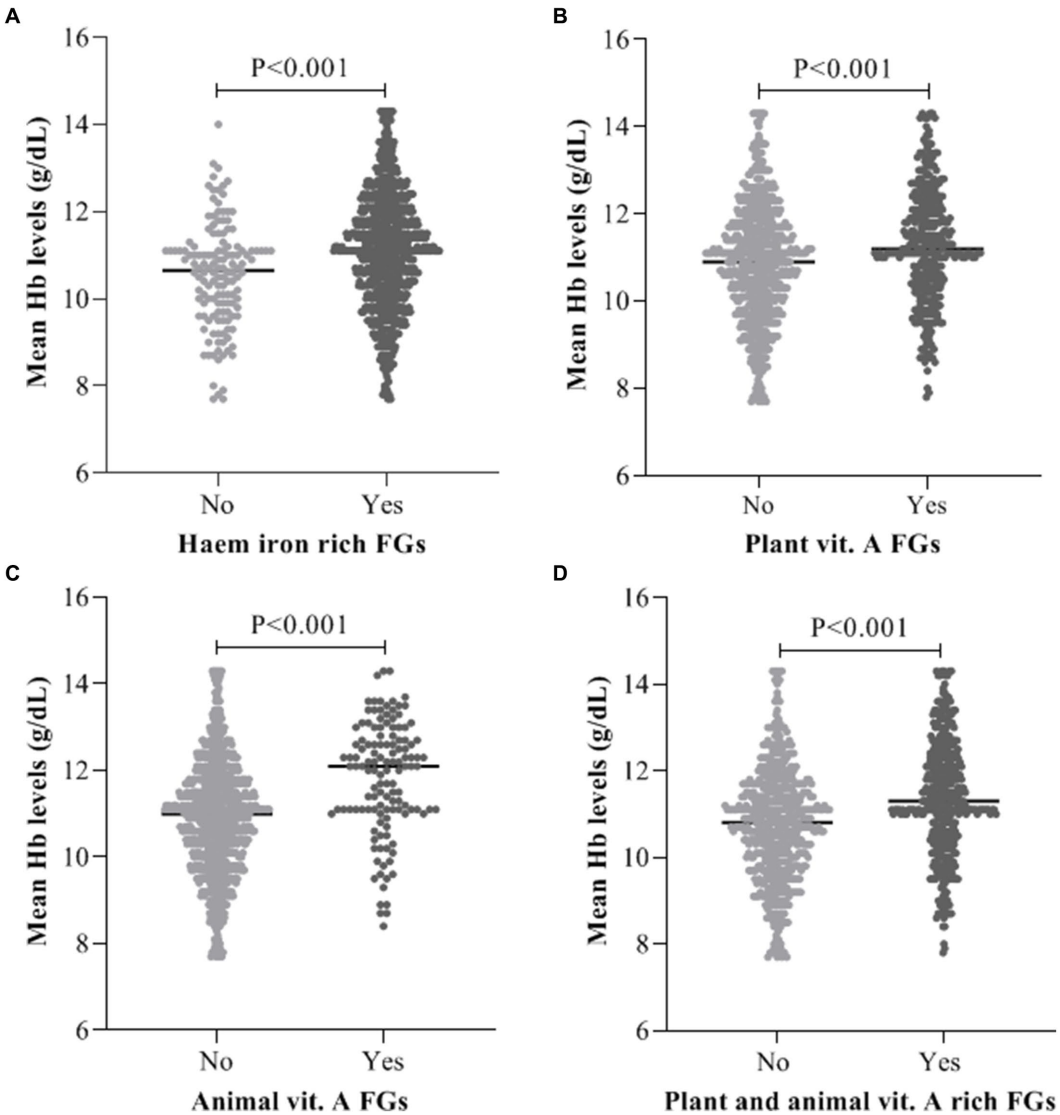
Average maternal Hb levels *Vs* intake of **(A)** Haem iron food groups, **(B)** Plant vitamin A food groups, **(C)** Animal vitamin A food groups, **(D)** Combine plant and animal vitamin A food groups.

As illustrated on Figure 1, mean maternal haemoglobin (Hb) levels was significantly (*p* < 0.001) high in women who consumed haem iron (11.08 ± 1.35), plant (11.25 ± 1.29) and animal foods rich in vitamin A (11.82 ± 1.30) when compared with their counterparts who did not consume haem iron (10.54 ± 1.19), plant (10.87 ± 1.34) and animal foods rich in vitamin A (10.88 ± 1.30; Figure 1).

## Discussion

In Cameroon, anaemia prevalence among women is still severe (≥ 40%) (16, 24, 25). This high prevalence rate may represent significant constraint for achieving the Global Nutrition Target endorsed by the World Health Assembly of halving anaemia prevalence among WRA by 2025 (20). This study therefore aimed to evaluate dietary micronutrient intake and their effect on haemoglobin levels of pregnant Cameroonian women.

In order to reduce the risk of anaemia during pregnancy, the WHO recommends a daily oral dose of 60 mg of iron along with 400 μg of folic acid throughout pregnancy and as part of the routine antenatal care services (37). In Cameroon, iron supplementation is the main strategy for anaemia control and prevention (16, 38). In addition, several studies have shown that iron-folic acid uptake during this critical period prevents maternal anaemia while reducing the risk of preterm labour, low birthweight, premature delivery, postpartum haemorrhage (39–41). The observed anaemia prevalence rate (40.9%) among study respondents in the study area despite uptake of iron-folic acid (71.5%) might be due to poor adherence, an aspect this study did not assess. Poor adherence to iron supplements may be as a result of inadequate supply of iron tablets, poor utilisation of prenatal health-care services, gastrointestinal discomfort accompanied with the drug, inability to purchase the tablet, forgetfulness, poor counselling by health care providers regarding the usefulness of the tablet as well as maternal knowledge and beliefs surrounding the tablet (42–44). Besides, this study further showed that combine uptake of iron-folic acid with a diet rich in haem iron or vitamin A food groups is more efficient in reducing the burden of anaemia than iron-folic acid taken alone.

Although diet holds great importance for maternal and neonatal health, inadequate proportions are often consumed most especially by women residing in low**-**and**-**middle income countries and study participants in the Mount Cameroon area were no exception (89.6%) (25, 45, 46). According to the WHO, the most common micronutrient deficiencies are; iron, vitamin A and iodine deficiencies (2, 47). In this study, 86.2% of the women consumed foods rich in iron specifically haem iron a day before the survey. Dietary iron is present in two forms that is haem iron, which is obtained from animal products such as meat, fish and poultry whereas non-haem iron is obtained from cereals, fruits and vegetables (36, 48, 49). Furthermore, it was observed in this study that consumption of haem iron was associated with increased haemoglobin levels of pregnant women. This finding is in line with observations from Jakarta (50), Ethiopia (51) and Pakistan (52). The increased haemoglobin levels among women who consumed meat, fish and poultry might be due to the fact that, foods rich in haem iron are absorbed from the gut with greater efficiency thus, making their iron content (the main component of haemoglobin) readily available for red blood cell production (51, 53).

Adequate vitamin A during pregnancy is essential for maternal and infant health (54, 55). Dietary vitamin A is available from two main sources that is, plants (provitamin A) and animals (preformed vitamin A) (55). Animal foods rich in vitamin A include; eggs, organ meat and dairy products while dark green leafy vegetables, vitamin A rich fruits and vegetables are plant foods rich in vitamin A (36, 56, 57). In this survey, 35.5 and 12.6% of the respondents enrolled consumed plant and animal food groups rich in vitamin A, respectively. The observed low intake of vitamin A animal food groups among study respondents might be due to the inability of the women to purchase eggs, organ meat and milk products. Furthermore, intake of foods rich in vitamin A was associated with maternal haemoglobin levels. Similar correlations have been described elsewhere (18, 58–60). Inadequate vitamin A intake is thought to cause anaemia through; reduction of the body’s immune response to infectious diseases which in turn leads to anaemia of infection, modulation of erythropoiesis and iron metabolism (6, 58, 61). Besides, vitamin A deficiency is known to increase the risk of iron deficient erythropoiesis and subsequently anaemia by altering absorption, storage, release and transport of iron to the bone marrow (62). This phenomenon might explain the low Hb levels observed among those who did not consume foods rich in vitamin A.

The current study had some limitations. Firstly, its cross-sectional nature could not establish the cause**-**and**-**effect relationship between dietary components and anaemia. In addition, this study did not measure biomarkers of micronutrient deficiency and other indicators of anaemia such as; mean corpuscular haemoglobin concentration (MCHC), mean corpuscular volume (MCV), reticulocyte count. In contrast, this study has as strength in its sample size as well as minimised recall bias by employing the use of the 24**-**h recall method to assess dietary diversity. Moreover, this study further demonstrates the effect haem iron and vitamin A rich food groups has on haemoglobin levels. Besides, this study sets the basis for future works determining the association and comparative influence of iron and vitamin A on Hb levels.

## Conclusion

Overall, the prevalence of anaemia (40.9%) was high despite adequate uptake of iron supplement (71.5%). Moreover, dietary diversity was inadequate (89.6%). In addition, anaemia prevalence rate was significantly (*p* < 0.05) lower in women who took IFA coupled with a diet rich in haem iron (38.5%) and vitamin A (29.0%). Furthermore, mean haemoglobin levels were significantly (< 0.001) higher in women who consumed haem iron (11.08 ± 1.35) and vitamin-A (11.34 ± 1.30) rich foods a day before the survey when compared with their respective contemporaries who did not. Thus, apart from focusing on iron**-**folic acid supplementation alone to curb the burden of maternal anaemia, public health authorities and health care givers should improve maternal nutritional awareness on the importance of a diversified diet as this would in turn enhance uptake of foods rich in haematopoietic nutrients thereby reducing anaemia prevalence rate.

## Data availability statement

The original contributions presented in the study are included in the article/supplementary material, further inquiries can be directed to the corresponding author.

## Ethics statement

The studies involving humans were approved by Institutional Review Board (IRB), Faculty of Health Science, University of Buea, Cameroon. The studies were conducted in accordance with the local legislation and institutional requirements. Written informed consent for participation in this study was provided by the participants’ legal guardians/next of kin.

## Author contributions

VJ: Formal analysis, Conceptualization, Data curation, Investigation, Writing – original draft, Writing – review & editing. JA: Formal analysis, Validation, Writing – review & editing. DS-F: Formal analysis, Validation, Writing – review & editing. GT: Formal analysis, Validation, Writing – review & editing. HK: Conceptualization, Supervision, Validation, Writing – review & editing. JA-K: Conceptualization, Supervision, Validation, Writing – review & editing.
